# Biochemical properties of lactic acid bacteria for efficient silage production: an update

**DOI:** 10.3389/fmicb.2025.1581430

**Published:** 2025-08-26

**Authors:** Muhammad Faheem Akhtar, Chai Wenqiong, Muhammad Umar, Wang Changfa

**Affiliations:** ^1^Research Institute of Donkey High-Efficiency Breeding and Ecological Feeding, College of Agronomy and Agricultural Engineering, Liaocheng University, Liaocheng, China; ^2^Faculty of Veterinary and Animal Sciences, Department of Animal Reproduction, Lasbela University of Agriculture, Water and Marine Sciences, Uthal, Pakistan

**Keywords:** lactic acid bacteria, recent advancements, silage, fermentation, lactic acid

## Abstract

Ensiling, a microbial-driven process employed for preserving fresh forage in both bio-refineries and animal production, triggers significant biochemical transformations. These changes have spurred the exploration of novel silage additives, with a particular emphasis on the potential of microbial strains that exhibit superior biopreservation capabilities. Lactic acid bacteria (LAB) species have gained widespread recognition for their diverse applications as additives in the fermentation of crops and forage biomasses during ensiling. Nonetheless, recent variations in silage quality might be attributed to a lack of comprehensive information on the gene expression and molecular mechanisms of the microbiota involved in silage production. Contemporary research efforts have been directed toward uncovering nutrient-rich animal feed solutions through enhanced LAB inoculants. This review aims to shed light on the role of LAB inoculants in silage production and the modern biotechnological methods, including metabolomics, proteomics, metagenomics, genomics, transcriptomics, and genetic manipulation. These powerful tools are instrumental in the identification, enhancement, and development of high-performance LAB strains. Additionally, the review outlines emerging trends and prospective developments in LAB advancement for the enhancement of silage, which holds significant promise for breakthroughs in sustainable agriculture and improved animal feed production.

## Introduction

Ensiling is a process, whereas ensilage is a product and is often referred to as silage. Animal feed production and biorefineries both use silage. Forage biomass, crop waste, and other industrial or agricultural byproducts can all be used to make it. When regular feed supplies are scarce, these materials are preserved by artificial or natural acidification, stored in an oxygen-free environment, and are frequently utilized as animal feed (Okoye et al., 2023). Fermenting plant biomass, typically with a moisture content of more than 50% produces silage. Compared to dry forages, it helps minimize nutrient loss during harvest and storage and enables more rapid and effective processing, which is why dairy farmers continue to choose it (Grant and Adesogan, 2018; Okoye et al., 2023). For instance, during periods of poor pasture growth or when pasture conditions are inappropriate for feeding animals, forage or crop biomasses are stored as alternative feed sources (Fabiszewska et al., 2019).

It has led to a notable rise in the demand for animal protein and renewable resources. As a result, the industries of forage and livestock are constantly pushing the need for forage, with the demand for animal protein expected to potentially double by 2030 (Alexandratos and Bruinsma, 2012; Giller et al., 2021). Forage preservation techniques that can both satisfy the growing demand and save the environment are therefore becoming more and more necessary. This is particularly crucial in humid locations where it can be difficult to preserve dry fodder (Font-i-Furnols et al., 2009; Nozière et al., 2014; Lara et al., 2018).

The ensiling process is a crucial biological process that results from natural fermentation under anaerobic conditions. It begins after forage crops are harvested at peak maturity. The process involves chopping the forage, placing it into a silo, compressing it to remove air and dust, and then storing it for future use as feed (Jones et al., 2004). A complex interplay of technological and biological elements affects silage quality. Lactic acid bacteria (LAB) break down plant biomass into lactic acid and other useful organic acids during fermentation. By fermentation, the pH is lowered to levels that stop dangerous spoiling microorganisms from growing (Adesogan and Newman, 2010; Yitbarek and Tamir, 2014). Initially, it was suggested that lactic acid production is mainly associated with non-spore forming bacteria, commonly known as LAB. This connection is due to their fermentative nature, with a strong ability to convert various carbohydrates into lactic acid. In food-related biotechnology, LABs hold a crucial role because of their safety for human and animal consumption, metabolic versatility, and adaptability to diverse ecological environments. As a result, they have gained interest ininnovative uses, including large-scale industrial fermentation (Okoye et al., 2022). LABs are commonly used for preserving the quality of ensiled forages (Oliveira et al., 2017a).

**Figure 1:**
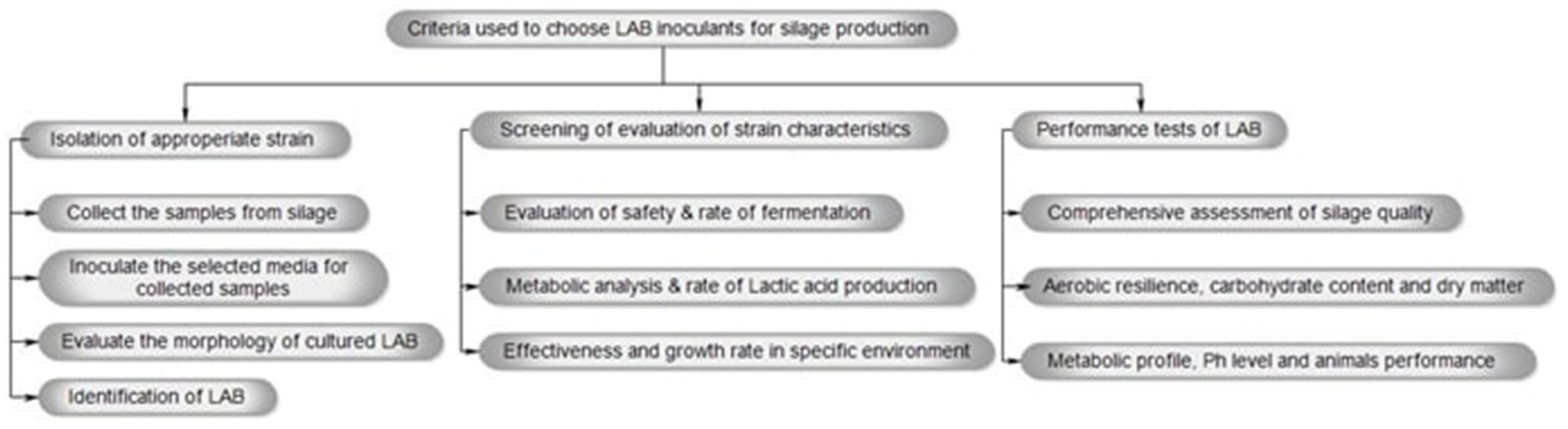
The criteria used to choose LAB inoculants for silage production involve isolating and screening appropriate strains, followed by testing their performance to assess their impact on the overall quality of the silage.

Figure 1 depicts the process for selecting LAB inoculants for silage production, which includes isolating and screening suitable strains and evaluating their performance to determine their effect on the overall quality of the silage. Additionally, the LAB species frequently used in silage production belong to the genera *Enterococcus, Lactiplantibacillus, Pediococcus, Lactococcus, Weissella, and Bacillus* (Ellis et al., 2016). Because of their potential to improve animal and human well-being, LABs are currently being used in modern, environmentally friendly agriculture with great enthusiasm (Amaral et al., 2020). Furthermore, it is often acknowledged that LAB inoculants are a dependable and efficient way to maintain fresh feed in animal production as well as bio-refineries. However, recent variations in silage quality may be related to a limited understanding of gene expression and the molecular mechanisms of the microbiota engaged in the silage production process (Wang et al., 2020). The development of modern biotechnological methods, such as next-generation sequencing (NGS) technology, has enabled the discovery of many promising mutants (engineered strains). Furthermore, these mutants have been successfully integrated into beneficial microorganisms, resulting in improvements in silage quality (Liu et al., 2015; Kröber et al., 2016; Horinouchi et al., 2017; Wang et al., 2017).

Recent advancements in biotechnology have made it possible to use meta-genomic sequencing to analyze the DNA of microbe in silage through various molecular techniques (Huws et al., 2015; Bao et al., 2016; McAllister et al., 2018). These methods have provided new understanding of the complexities of microbial roles in improving ensiling. They have clarified the importance of LAB populations in the silage process and how using LAB inoculants can promote microbiomes that are more conducive to producing high-quality silage (McAllister et al., 2018). Enhancement of strains can also lead to the development of high-performing strains for silage production and preservation. These efforts have been successful in locating and producing novel LAB strains with particular desired characteristics (Wang et al., 2018). This review describes the function of LAB in silage production and preservation. In the framework of sustainable agriculture, it highlights contemporary molecular methods for improving LAB and offers recommendations for future paths for developing silage processing technology.

## Inoculants of lactic acid bacteria (LAB)

The search for novel silage additives has been made easier by a clear and understanding of the biochemical changes that occur during ensiling, with a focus on unique strains that display higher efficiency (Xu et al., 2019). Several silage additives-derived from plant enzymes, microbes, or field-associated factors-can help reduce inevitable losses. Formic acid, sorbic, acetic, propionic, benzoic, and their salts are examples of chemical additions. Cellulase and homo and heterofermentative LAB are examples of biological additives whose effects have been thoroughly investigated (Kim et al., 2021; Soundharrajan et al., 2021). These silage additives are applied to forage or crop biomass during ensiling to enhance the fermentation process. The functions include reducing dry matter (DM) losses, preventing aerobic deterioration during feed distribution, improving the overall hygienic quality of the silage, controlling the secondary fermentation, enhancing the aerobic stability, boosting the nutritional value of the silage, inhibiting the pathogen activity, and ultimately increasing the animal production. Additionally, these additives are anticipated to offer farmers benefits that outweigh their costs (Yitbarek and Tamir, 2014). For silage inoculation, however, biological options particularly LAB are favored above other additives. This favor stems from their advantageous impacts on dry matter recovery, fermentation properties, and animal performance, as well as their safety, practicality, non-corrosiveness, and environmental friendliness (Ni et al., 2015a). Table 1 outlines the components of LAB inoculants and other additives used in silage production, along with their effects on silage preservation.

**Table 1:** The effectiveness of LAB (Lactic Acid Bacteria) inoculants in the production of silage.

Silage type	LAB starter culture	Inoculation level (CFU/g)	Fermentation period (days)	Dry matter (g/kg FW)	Contemporary methods employed	Effects on the quality of the silage	References
Rice straw	*Limosilactobacillus reuteri, L. salivarius, L. plantarum, L. brevis, S. bovis*	1 × 10^6^	30	850.2	16 S rRNA, Real-time PCR, HPLC	Reduced methane generation, improved silage quality, microbial populations, and parameters of rumen fermentation	Amaral et al. (2020)
Alfalfa	*Lactiplantibacillus*. *pentosus, Lactiplantibacillus. pentosus* + *L. brevis* + *P. acidilactici*	1 × 10^6^	90	331.4 and 435.2	16 S rRNA, HPLC	Exhibited higher remaining water-soluble carbohydrates (WSC) content and the lowest pH	You et al. (2021)
Sorghum	*L. casei*	1 × 10^5^	30	259.7	HPLC	Altered in-vitro digestibility and emission of methane	Kaewpila et al. (2021)
Ryegrass	*L. plantarum, Lc. Lactis*	1 × 10^6^	60	243	NA	Enhanced the degradability of dry matter and organic content in vitro	Huyen et al. (2020)
Elephant grass	NA	NA	NA	NA	NA	Facilitated reduced yeast populations, negligible levels of butyric acid, and improved aerobic stability	Amaral et al. (2020)
Whole-crop corn	*L. plantarum, P. pentosaceus*	NA	45	NA	HPLC	Suppressed the proliferation of filamentous fungi and yeasts, leading to a decrease in silage pH	Amaral et al. (2020)
Whole-crop corn	*E. faecium*, *L. plantarum*, *L. brevis*	1 × 10^5^	100	323	GC–MS	Increased digestible energy, higher metabolizable energy concentration, enhanced aerobic stability, elevated dry matter intake, improved weight gain, and enhanced feed conversion ratio	Acosta Aragón et al. (2012)
Alfalfa	*L. plantarum, L. pentosus, P. pentosaceus*	1 × 10^5^	56	368	NA	Effectively managed entero-bacteria and mold populations, resulting in improved chemical properties of the silage, including an elevated index of in-vitro dry matter digestibility	Nascimento Agarussi et al. (2019)
Ryegrass	*L. plantarum* + *Lc. lactis* + *L. buchneri*	1 × 10^5^ 1 × 10^6^	210	450	GC–MS	Elevated milk production, although the effects on animal performance were limited for both short-term and long-term inoculation of grass silage	Ellis et al. (2016)
Whole-crop corn	*L. buchneri, P. pentosaceus*	1 × 10^5^ 4 × 10^5^	120	310–390	Real-time PCR, HPLC	Enhanced aerobic stability and fermentation characteristics	Schmidt and Kung Jr (2010)
Whole-crop corn	*L. plantarum, L. buchneri*	6 × 10^10^ 2 × 10^10^	240	331	HPLC	Improved resistance to aerobic conditions and fermentation characteristics	Hu et al. (2009)
Corn stalk	*L. plantarum*	5 × 10^10^	60	450	HPLC	Improved enzymatic breakdown of corn stalk silage	Li et al. (2019)
Mixed tall fescue, meadow fescue	*L. plantarum, L. plantarum* + *L. buchneri*	1 × 10^6^	60	179	HPLC	Enhanced the resistance to aerobic conditions and the dry matter content	Guo et al. (2013)
Corn stover	*L. plantarum*	1 × 10^5^	42	174.8	16 S rRNA, PacBio SMRT, HPLC	Altered the composition of the microbial community, improving silage fermentation	Yan et al. (2019)
Corn stover	*Lc. Lactis, L. buchneri, L. brevis, L. plantarum, L. rhamnosus*	1 × 10^5^	120	428	HPLC, GC–MS/MS	Enhanced resistance to aerobic conditions and decreased the occurrence of various mycotoxins in the silage	Gallo et al. (2021)
Corn straw	*L. plantarum, L. buchneri, L. farraginis*, *P. acidilactici*	1 × 10^6^	100	454	HPLC	Reduced dry matter loss and lowered yeast counts throughout the entire ensiling perio	Costa et al. (2021)
Alfalfa, corn straw	*P. pentosaceus, P. acidilactici*, *L. acidophilus, L. plantarum*	2 × 10^5^	59	168, 175	GC–MS	Enhanced silage qualities and fiber breakdown in alfalfa, with no observable impact on corn silage.	Chilson et al. (2016)
Stylo, rice straw	*L. plantarum*	1 × 10^6^	30	270, 373	16 S rDNA, Illumina HiSeq, GC–MS, HPLC	Modified the odor through adjustments in the microbial populations within the silage	Zhao et al. (2021)
Sugar beet pulp + rice straw	*L. delbrueckii, L. bulgaricus, L. acidophilus*	1 × 10^11^	14	300	HPLC	Improved the feed consumption, milk production, nutrient absorption, and plasma metabolite levels in dairy cows	Wang et al. (2022)

The composition of LAB in silage can vary significantly, making it crucial to carefully select and apply inoculants to achieve high-quality silage (Oliveira et al., 2017a; Drouin et al., 2019). This screening procedure assesses the ability to produce organic acids, the capacity of proteins to degrade, the growth rates under various pH and temperature conditions, and the overall performance in a range of assays. The natural habitat of the inoculant in plants, its capacity to flourish in recently chopped or chopped plant material, its resistance to bacteriophages, its compatibility with co-cultures, its genetic stability, its resilience to environmental stress, and its capacity to impede the growth of molds and yeasts are additional considerations when choosing an inoculant (Carvalho et al., 2021). Additionally, the fermentation of plant biomass is greatly affected by the ensiling environment and the LAB’s ability to rapidly adapt and utilize the available nutrients (Amaral et al., 2020; Guan et al., 2020; Carvalho et al., 2021). The two main forms of LAB inoculants, i.e., homo-fermentative and hetero-fermentative LAB are the focus of silage production when it comes to natural plant biomass fermentation.

### Homo-fermentative LAB

Homofermentative LABs are among the oldest and most commonly used inoculants in silage production (Fabiszewska et al., 2019). The capacity of homofermentative LAB strains to produce large amounts of lactic acid during fermentation makes them popular choices for silage production. Examples of these strains include *Pediococcus, Streptococcus, Enterococcus,* and *Lactiplantibacillus* (Kim et al., 2021). For legume silages, homofermentative LAB inoculants are frequently chosen because they efficiently lower dry matter (DM) losses by producing greater amounts of lactic acid. Notably, the capacity of *Lactiplantibacillus plantarum*, one of the most popular homo-fermentative LAB species, to rapidly decrease pH, suppress harmful microbes, and maintain plant proteins has been well-documented (Guo et al., 2013; Xu et al., 2017; Zhang et al., 2020; Bai et al., 2021).

**Figure 2:**
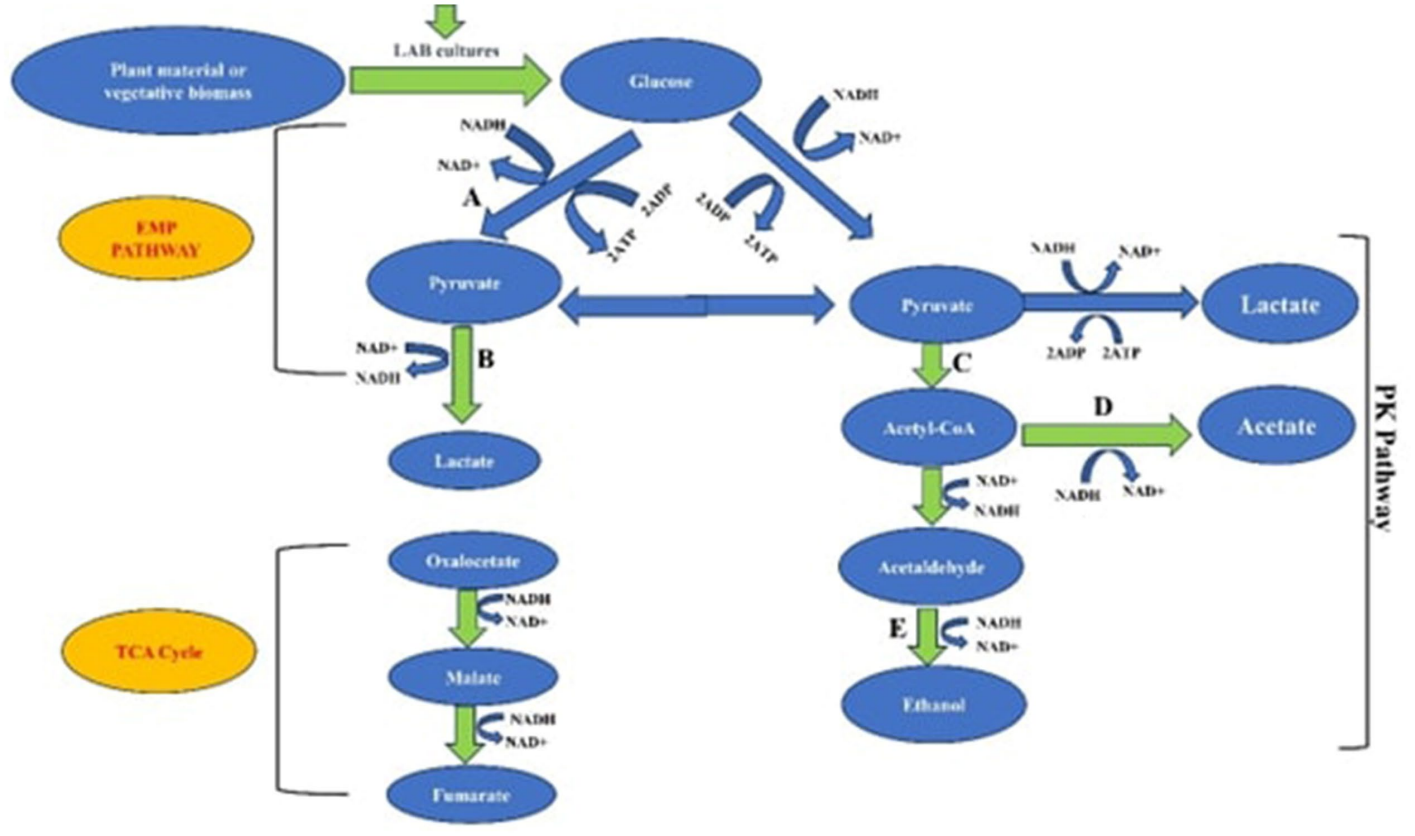
Metabolic pathways employed by LAB inoculants in the synthesis of organic acids during the silage production process. Blue arrows represent homofermentation, while yellow arrows represent heterofermentation. Key pathways include the Embden-Meyerhof pathway (EMP), phosphoketolase pathway (PK), tricarboxylic acid cycle (TCA), and enzymes like pyruvate kinase **(A)**, lactate dehydrogenase **(B)**, oxidative dehydrogenase **(C)**, acetyl-CoA synthetase **(D)**, and alcohol dehydrogenase **(E)**.

This is because of the strong and distinctive probiotic qualities of *L. plantarum,* which include its adaptability, strong resistance to bile and acidic environments, and capacity to inhibit pathogenic microbes (Yadav et al., 2016). However, incorporating *P. pentosaceus* into the silage resulted in higher dry matter digestibility compared to using *Lactiplantibacillus plantarum* (Nascimento Agarussi et al., 2019). Additionally, various *Pediococcus* species have been proposed for probiotic use because of their antioxidant, anti-inflammatory, detoxification, and lipid-lowering properties (Jiang et al., 2021). It is noteworthy that studies have demonstrated a 3 to 5% improvement in animal performance with homofermentative LAB. When compared to the use of heterofermentative LAB, they yield a higher dry matter recovery of about 2 to 3%, which is attributed to their ability to produce lower levels of ethanol, acetic, and butyric acids (Muck et al., 2018). Figure 2 explains the metabolic pathways used by LAB inoculants to produce organic acids during the process of silage production.

*Leuconostoc, Oenococcus, Weissella,* and *Lactiplantibacillus* are the genera that include the majority of heterofermentative LAB. In addition to lactic acid, these kinds of LAB also create acetic acid, ethanol, and carbon dioxide from hexoses (Muck et al., 2018). They are identified as the largest population of LAB capable of producing significant quantities of acetic acid by converting lactic acid during fermentation (Ni et al., 2016). Since the production of moderate acetic acid can inhibit the growth of yeasts and molds causes spoilage after exposure to air. Moreover, heterofermentative LAB inoculants are valuable for reducing dry matter losses, improving aerobic stability, and minimizing losses during feed distribution (Muck et al., 2018). Nevertheless, the efficiency of just a small number of *Lactiplantibacillus* species in the formation of silage has been investigated. *Lentilactobacillus buchneri* is one among these, and *Lentilactobacillus kefiri, Lentilactobacillus diolivorans, Levilactobacillus brevis, Lentilactobacillus hilgardii, and Furfurilactobacillus parafarraginis* are also present to a lower degree (Muck et al., 2018). *L. buchneri* is an effective silage inoculant for producing high-quality silage with minimal dry matter loss. This is due to its strain’s high tolerance to acids and bile, antimicrobial activity, and greater resistance to heating during feed distribution (Romero et al., 2017). Numerous studies have revealed the effect of *L. buchneri* on silage quality depends on the specific strain and the dosage applied (Kung et al., 2018; Muck et al., 2018). Furthermore, because *L. hilgardii* can produce acetic acid and withstand long-term ensiling, its application reduced yeast populations and enhanced the aerobic stability of silage (Ferrero et al., 2021). Nevertheless, the production of a variety of organic acids is a distinctive feature of heterofermentative LAB inoculants (Ning et al., 2017; Puntillo et al., 2020; Soundharrajan et al., 2021).

### Additional properties of LAB

Historically, lactic acid bacteria have been employed in industrial fermentation as valuable chemical producers and starter cultures. Lactic acid is a primary product and is particularly notable as one of the most in-demand chemicals. However, LAB also shows promise as a candidate for producing a range of other substances, including sweeteners, bacteriocins, vitamins, lignocellulose enzymes, exopolysaccharides (EPS), and more (Tarraran and Mazzoli, 2018). These substances are known for their various functions, including bio-preservation and refining the nutritional quality of the silage. This enhancement broadens the applications of LAB inoculants that produce specific vitamins in fermented products (Wu et al., 2017). In addition to the previously mentioned pathways, LAB is used in various methods to produce additional metabolites such as diacetyl, L-alanine, mannitol, vitamins, sorbitol, and EPS. These metabolites contribute to improving the nutritional profile, reducing harmful compounds, extending the shelf life, and enhancing the flavor of fermented products. Vitamins are the complex organic compounds needed in small amounts as supplements and additives to play a crucial role in this process (Averianova et al., 2020). Food products become more nutritious when LAB fermentation produces vitamins like riboflavin, folic acid, vitamin C, and pyridoxal (Florou-Paneri et al., 2013). It has been shown that LAB, which includes *Lactobacillus acidophilus*, *Limosilactobacillus fermentum, L. plantarum*, and *Ligilactobacillus lactis*, increases the nutritional value of fermented foods.

This suggests their ability to enhance the nutritional value of food products without the need for additional fortification (Thakur et al., 2016). Furthermore, these traits can be explored as a substitute solution to the issues faced with aerobically unbalanced silage, which can develop musty or moldy odors and suffer from nutritional value due to yeast and mold growth, as reported by Kung et al. (2018). In addition, the use of *L. buchneri,* which produces 1,2-propanediol, improved the corn silages by aerobic stability and played a key role in supporting dairy cow health by helping to prevent ketosis (Huang et al., 2021). Furthermore, several LAB species can produce EPS, such as *Streptococcus thermophilus, Limosilactobacillus reuteri, Lacticaseibacillus casei*, and *L. plantarum*. These compounds are used in fermented foods as food additives in the industry due to their effects on rheological and textural properties. EPS gives LAB a competitive edge by aiding surface colonization, biofilm dominance, and improving the quality of fermented plant-based products like silages. For instance, silage inoculated with EPS-producing *P. pentosaceus* showed higher antioxidant as well as antibacterial activities, primarily due to its ability to scavenge hydroxyl free radicals and completely inhibit the growth of *Staphylococcus aureus* (Fan et al., 2021). Additionally, bacteriocins are antimicrobial substances produced by LAB, derived from proteins, polypeptides, or protein complexes.

**Figure 3:**
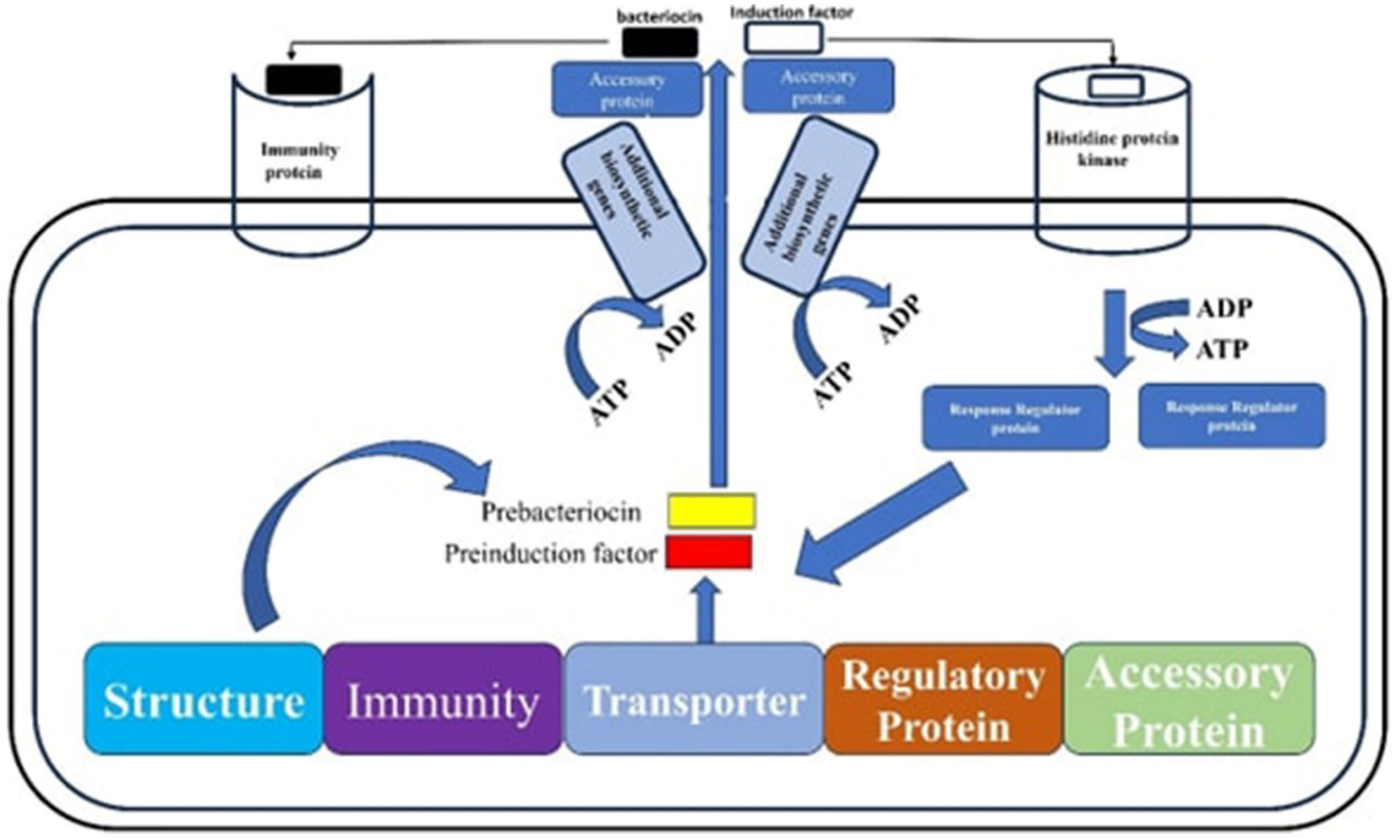
The metabolic pathways involving bacteriocins start with the formation and modification of prebacteriocin BacA into BacB and BacC. This is followed by its processing into probacteriocin BacP and its translocation via the ABC-transporter BacT. Bacteriocin synthesis is then regulated by a signal sensor histidine protein kinase (HPK), gene transcription is facilitated by the response regulator (RR) protein and an inducer peptide (IP). Finally, immunity is provided by immunity proteins (IMP) such as BacL.

These are frequently employed as preservatives in the manufacture of silage because they successfully stop the growth and spread of harmful microbes (Wang et al., 2022). For example, introducing class IIa bacteriocin-producing *Lactobacillus delbrueckii* into alfalfa silages led to a reduction in mold and yeast populations, improved the overall fermentation quality of the silage, and significantly enhanced its aerobic stability compared to the commonly used inoculant, *L. plantarum* (Zhang et al., 2020). Additionally, some LABs can produce a variety of lignocellulolytic enzymes, such as cellulases, hemicellulases, oxidases, peroxidases, proteases, chitinases, mannanases, amylases, and pectinases. These enzymes function as biocatalysts, dissolving lignocellulose, an essential part of plant biomass, into its constituent parts (Chukwuma et al., 2020). To enhance the ensiling process for forages used in cattle feed, for instance, LAB that produce lignocellulolytic enzymes, such as *Enterococcus species* and *Paenibacillus sp*ecies have recently been found in guinea grass. These LAB strains released hemicellulose-breaking enzymes such as xylanases, endo-glucanases, esterases, and arabinofuranosidase, which were particularly useful at breaking down oligosaccharides and using xylose (Díaz-García et al., 2021). Additionally, under optimal growth conditions such as appropriate temperature and pH, *B. coagulans* strains have been found to produce soluble thermophilic cellulases. These cellulases are particularly effective in environmental conditions, whereby lignocellulosic materials are often broken down by fungal enzymes into fermentable sugars. This enzymatic activity improves the nutritional quality of the biomass and encourages greater silage consumption by animals (Aulitto et al., 2017). Figure 3 shows the metabolic pathways involving bacteriocins.

### Effects of LAB inoculations through collaboration

Numerous studies indicate that mixing various LAB strains can synergistically improve silage quality (Tian et al., 2017). Recent studies have shown successful results from combining LAB strains to improve silage quality (Kim et al., 2021). Historically, homofermentative LAB species have been employed to speed up the first phases of ensiling to increase the quality of the silage. The process is finished when water-soluble carbohydrates (WSC) quickly ferment into lactic acid, which causes the pH to drop quickly. The advantages of preservation are, however, limited since, in aerobic conditions, microbes can quickly convert them into lactic acid. Thus, the primary benefit of these strains is the pH decrease they offer.

Heterofermentative LAB strains, on the other hand, generate a lot of acetic acid, which helps stop aerobic spoiling. Combining LAB inoculants, including *L. buchneri* with other traditional homofermentative strains, has shown to be helpful for a variety of crops and forage biomass due to their effectiveness. This combination guarantees rapid pH decline and efficient early fermentation, which is followed by heterofermentative LAB’s synthesis of acetic acid from lactic acid (Borreani et al., 2018). Recent research has highlighted the advantages of using a variety of LAB inoculants alongside *L. buchneri* for preserving high-quality silage. This implies that silage quality can be specifically impacted by LAB strains with varying fermentation characteristics. Mixtures of *L. plantarum, Lacticaseibacillus casei, Pediococcus acidilactici, Pediococcus pentosaceus, Enterococcus. faecium, Lactococcus lactis*, and *Bacillus subtilis* are among the notable combinations. Similarly, *Pediococcus pentosaceus* with *L. plantarum, L. plantarum* with *E. faecium* are noteworthy (Huisden et al., 2009; Adesogan and Newman, 2010; Acosta Aragón et al., 2012; Addah et al., 2014; Ellis et al., 2016). Additionally, using a combination of the two heterofermentative strains, *L. hilgardii* and *L. buchneri*, significantly improved the aerobic stability of corn silage compared to the combination of *Pediococcus pentosaceus* and *L. buchneri* (Da Silva et al., 2021). During the silage-making process, LAB strains with various functionalities have been effectively mixed with either homofermentative or heterofermentative LAB. For instance, bacteriocin-producing *Lactobacillus delbrueckii* combined with homofermentative *L. plantarum* resulted in a notable decrease in the growth of mold and yeast in alfalfa silage (Li et al., 2020). Likewise, combining homofermentative *L. plantarum* with cellulase-producing *Bacillus pumilus* strains improved the overall quality of alfalfa silage (LI et al., 2018).

**Figure 4:**
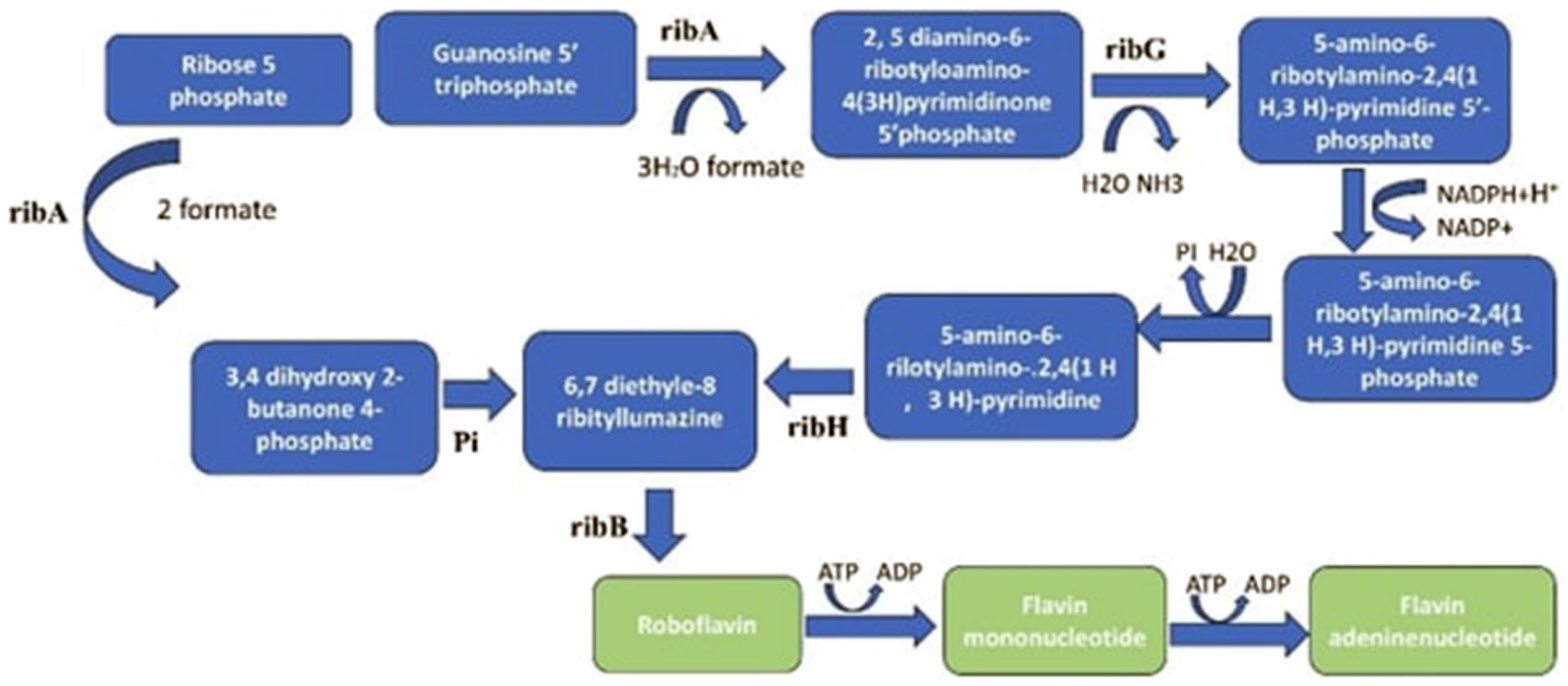
The metabolic pathways for riboflavin biosynthesis begin with guanosine-5′-triphosphate (GTP) and ribulose-5-phosphate (Ru5P). These substrates are processed by the enzymes GTP cyclohydrolase II and 3,4-dihydroxy-2-butanone-4-phosphate synthase (DHBPS), which are encoded by the genes ribA, ribB, ribG, and ribH. GTP is converted into 2,5-diamino-6-ribitylamino-4(3 H)-pyrimidinone 5′-phosphate, while Ru5P is converted into 3,4-dihydroxy-2-butanone 4-phosphate. The subsequent enzymatic reactions, involving pyrimidine deaminase, pyrimidine reductase, and lumazine synthase, culminate in the synthesis of riboflavin through the dismutation of two molecules of 6,7-dimethyl-8-ribityllumazine by riboflavin synthase.

Similarly, combining cellulase-producing *B. subtilis* with heterofermentative *L. buchneri* significantly influenced the ensiling process, resulting in enhanced nutritional value and improved quality of corn silage (Zhang, 2019). More advantages would be possible, though, if these combinations are further refined to fit varied ensiling circumstances, such as different plant biomass kinds and climates. In addition to preventing the growth of *Enterobacteria*, *Clostridium*, and other dangerous microbes, this would hasten the reduction of pH and dry matter losses and assist in better control of the initial active fermentation phase (Muck et al., 2018). Figure 4 shows the metabolic pathways responsible for riboflavin biosynthesis as follows.

### Metabolic routes of lab inoculants

LABs are important in several fermentation processes. Their physiology is quite simple, they exhibit metabolic variety, and their limited biosynthetic capacities are all significant for understanding their metabolic pathways. Furthermore, a comprehensive comprehension of their genetics, physiology, molecular biology, and biochemistry has improved the efficiency of their metabolic processes (Nuryana et al., 2019). LAB utilize various metabolic pathways, including glycolysis for sugar fermentation, lipolysis for fat breakdown, and proteolysis for protein degradation. These processes produce numerous beneficial metabolites, such as organic acids, bacteriocins, and EPS. As a result, LAB play a versatile role in improving, producing, and preserving of fermented foods and silage (Bintsis, 2018).

### Biosynthesis of organic acids

Understanding the metabolism of organic acids in LAB requires examining specific metabolic processes, particularly anaerobic fermentation, which generates various organic acids such as lactic, acetic, formic, succinic, and citric acids (Wang et al., 2022). For example, during ensiling, LAB uses glucose as a source of carbohydrates and uses glycolysis to turn it into pyruvate. After that, they use lactate dehydrogenase (LDH) and the phosphoketolase (PK) pathway to metabolize lactic acid (Kim et al., 2021). Additionally, glucose 6-phosphate is converted into CO_2_, ribulose 5-phosphate (Ru5P), and Nicotinamide Adenine Dinucleotide Phosphate (NADPH) via the pentose phosphate (PP) pathway.

LDH is a crucial enzyme in LAB, facilitating the conversion of pyruvate into lactic acid and regulating the metabolism of lactic acid through its stereospecificity (Wang et al., 2022). Whereas heterofermentative LAB also creates CO_2_ and ethanol in addition to lactic acid, homofermentative LAB primarily produce lactic acid as the only fermentation product. Furthermore, LAB has at least two citrate metabolic pathways via which it can create succinate, formate, and acetate. The first pathway reduces oxaloacetate and transforms citrate into succinate by using enzymes such as fumarase, fumarate reductase, and malate dehydrogenase. Pyruvate is metabolized into lactate, acetate, formate, and ethanol by the second route (Nuryana et al., 2019). Previous research has mainly focused on studying the combined effects of LAB that produce these organic acids, with limited comprehensive data on their metabolic interactions during the silage production process (Jiang et al., 2020).

### Production of bacteriocins

Bacteriocins are classified into four groups based on factors such as post-translational modifications, amino acid compositions, mechanisms of action, spectrum of activity, and molecular mass (Negash and Tsehai, 2020). Class I bacteriocins, also known as lantibiotics, are small peptides with a molecular weight under 5 kDa, including examples like lacticin, mutacin, subtilin, and nisin. Class II, or non-lantibiotics, consists of small peptides with a molecular weight under 10 kDa, such as pediocins and enterocins. Class III bacteriocins are heat-stable proteins with larger peptides exceeding 30 kDa, including caseicin and helveticin. Class IV bacteriocins, which contain non-protein components, are primarily made up of lipids or carbohydrates, with examples like *leuconocin Species* and *plantaricin Species* (Meade et al., 2020; Simons et al., 2020; Sharma et al., 2021). In LAB, bacteriocins are produced via distinct pathways that begin with the synthesis of pre-bacteriocin and end with a cleavage step that removes the leader sequence. This processing is essential for transporting the pro-bacteriocin across the cell membrane. The synthesis of bacteriocins involves several genes, usually arranged in an operon cluster to facilitate efficient production (Sharma et al., 2021). Moreover, the synthesis and secretion of bacteriocins involve signal transduction systems comprising three essential components: histidine protein kinase (HPK), regulatory protein (RR), and inducer peptide (IP).

### Production of riboflavin

Riboflavin production in LAB follows the riboflavin biosynthesis pathway (RBP), starting with guanosine-5-triphosphate (GTP) and ribulose-5-phosphate (Ru5P) (Averianova et al., 2020). The four genes ribA, ribB, ribG, and ribH encode the enzymes needed for this pathway, which is how riboflavin is synthesized from GTP and Ru5P (Sepúlveda Cisternas et al., 2018; Averianova et al., 2020). Although the order of these genes does not correspond to the sequence of enzyme activities, they are arranged within an operon. In the rib operon, ribG is the first gene in the transcriptional sequence and encodes a bifunctional enzyme with deaminase and reductase activities, whereas ribA, though not first in the operon, initiates riboflavin biosynthesis by encoding GTP cyclohydrolase II, which converts GTP into the initial pyrimidine intermediate. The last stage in the biosynthesis of riboflavin is catalyzed by lumazine synthase, which is generated by the last gene, ribH. The riboflavin synthase gene, encoded by the second gene, ribB, completes the pathway by generating riboflavin. The second gene, ribB, encodes riboflavin synthase, which catalyzes the final step of the pathway to produce riboflavin.

### Exopolysaccharides production

Exopolysaccharides (EPS) are produced by different LAB species, including *Streptococcus* and *Lactiplantibacillus*, each utilizing distinct biosynthetic pathways (Wang et al., 2022). Furthermore, the genes that produce EPS are usually grouped together in the producing organism’s genome. Therefore, for both metabolic and genetic techniques aiming at synthesizing this useful polymer, a detailed understanding of the intricacies of EPS formation and the mechanisms governing these activities is critically important (Schmid et al., 2015). LAB that produce EPS utilize three fundamental mechanisms (Andhare et al., 2014). Three main processes are involved in the synthesis of EPS in LAB. The first mechanism uses glucosyltransferases (GTs) to add sugar units one after the other to create repeating units. This pathway is dependent on Wzx and Wzy (key enzymes). The Wzx flippase is responsible for moving these units across the cytoplasmic membrane. The Wzy protein translocates the oligosaccharide units, polymerizes them into polysaccharides, and exports them to the cell surface. The second process is the transporter-dependent ABC (ATP-binding cassette) system that synthesizes capsular polysaccharide (CPS). Through the ABC transporter system, glucosyltransferases at the cytoplasmic face’s inner membrane assemble the CPS in this mechanism.

The third process, known as the synthase-dependent pathway, creates entire homopolymer strands across membranes and cell walls using a single synthase protein. This mechanism facilitates the transfer of monomeric repeating units without requiring the involvement of the Wzx flippase.

### Functions of LAB in the silage production process

In the past, ensiling was recognized for the creation of silage with either a “sweet” or “sour” flavor (Bernardes et al., 2018). Similarly, more than 90% of forage crops such as maize, sorghum, grasses, legumes, and wheat are cultivated and processed as silage locally (Mohd-Setapar et al., 2012). The production of high-quality silage involves a key biochemical process: spontaneous fermentation by LAB in an aerobic environment. This process preserves various nutritious forage crops, enabling their efficient storage and protecting their quality from the point of proper harvesting (McAllister et al., 2018; Fabiszewska et al., 2019).

Three primary objectives are achieved by introducing LABs into the process: (1) to inhibiting the growth of spoilage bacteria, (2) lowering the pH of the silage, and (3) enhancing the dry matter recovery (Ren et al., 2021). However, the critical biochemical changes required for effective silage fermentation with LAB inoculants include the removal of oxygen, the fermentation process, an appropriate concentration of water-soluble carbohydrates (WSC), enhanced pH reduction, decreasing the buffering capacity, proper forage wilting, maintenance of suitable temperature conditions, and timely feed-out.

### Aerobic phases

LAB ferments plant sugars in recently cut material during this period, releasing heat, water, and carbon dioxide. Simultaneously, these sugars are used by aerobic microorganisms on the surface of the plant, including bacteria, yeast, and molds, which improve respiration. The quick development of mold and yeast during this early stage can raise the possibility of heating and spoiling. Sustaining silage quality and managing hazardous microorganisms need efficient processing of the silage, which includes LAB inoculation (Susanti et al., 2022). Moreover, the use of mixed inoculants on plant biomass inhibits the activity of yeasts, molds, and other aerobic bacteria, which would normally consume the lactic acid efficiently produced by homofermentative LAB (Muck et al., 2018).

### Fermentation process

**Figure 5:**
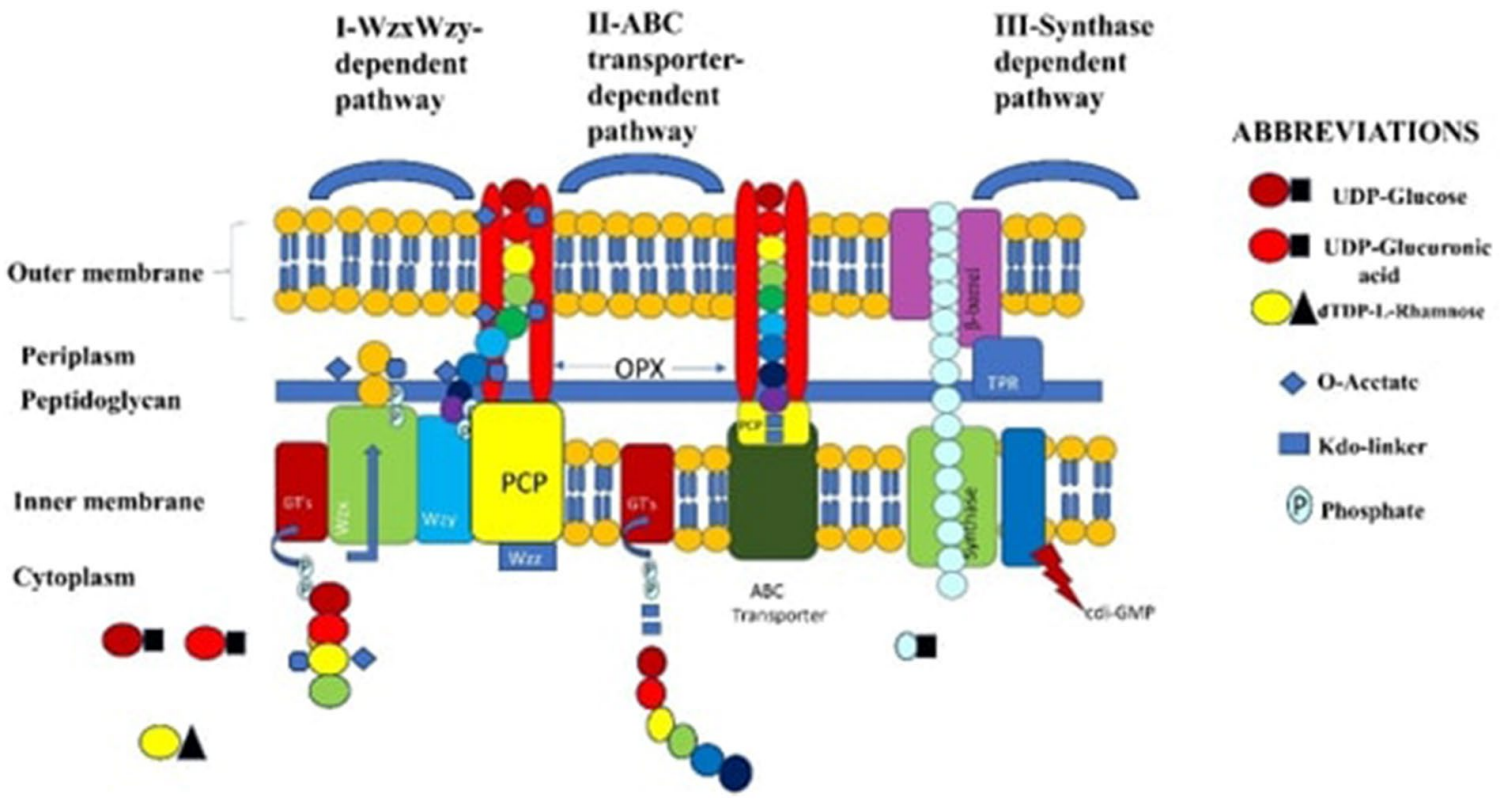
Metabolic pathways for EPS (exopolysaccharides) are primarily categorized into three intracellular routes: (I) The Wzx/Wzy-dependent pathway, where glycosyltransferases (GTs) assemble the repeating unit of the polysaccharide. (II) The ABC transporter pathway, which constructs the polysaccharide chain and then transports it across the cell wall and membranes. (III) The synthase-dependent pathway, where the synthase complex polymerizes and transports the entire polysaccharide across the cell membrane.

The anaerobic fermentation phase encompasses a series of various bacteria that engage in the fermentation of sugars derived from plants (Gollop et al., 2005). Bacteria abound on the surface of plants, causing spontaneous anaerobic fermentation of plant material. However, the amount and kinds of LAB present in the biomass of the plant determines the rate and effectiveness of this fermentation, particularly about pH decrease. These LAB have the ability to inhibit the growth of undesirable microorganisms (LI et al., 2018). One of the most effective methods for preserving silage is to enhance fermentation with LAB. These bacteria protect forage materials from spoilage by producing beneficial organic acids and antifungal agents, such as bacteriocins (Santos et al., 2013). Furthermore, because homofermentative LAB species only produce lactic acid, they are better at reducing the pH of silage. On the other hand, acetic acid produced by heterofermentative LAB can obstruct the pH drop during this phase (Schmidt and Kung, 2010; Oliveira et al., 2017b). Here above Figure 5 explains metabolic pathways for EPS.

### Equilibrium phase

Due to the influence of LAB, ensiled forages enter the storage phase, often called the stability stage. During this time, when the silo is properly sealed, biological activity remains minimal (Borreani et al., 2018). During this stage, silage treated with LAB maintains a constant pH level, generating an acidic environment that inhibits microbial growth. As long as the silo is sealed, dangerous species like Bacilli and Clostridium may persist as spores (LI et al., 2018). Furthermore, numerous studies have highlighted LAB’s ability to maintain a low pH in silage, produce beneficial organic acids and byproducts, and reduce the presence of harmful pathogens during this period (Bai et al., 2021; Kim et al., 2021). After the silo is opened, silage needs to be used right away to avoid aerobic spoiling. Yeasts begin to break down the organic acids generated by LAB during fermentation as soon as oxygen enters the ensiled feed. This procedure can cause the silage to further deteriorate since it increases pH and rekindles aerobic activity (Peters and Hoffmann, 2010; Han et al., 2014). The kinds and concentrations of organic acids and other metabolites that LAB produces during fermentation have an impact on the aerobic stability of silages during feed-out. LAB produces organic acids that are more hazardous to yeasts and molds than lactic acid, including butyric acid, acetic acid, and propionic acid. Therefore, compared to silages inoculated with heterofermentative LAB, those treated with highly effective homofermentative LAB frequently exhibit lower aerobic stability (Borreani et al., 2018).

### Effect of LAB inoculants silage quality

**Figure 6:**
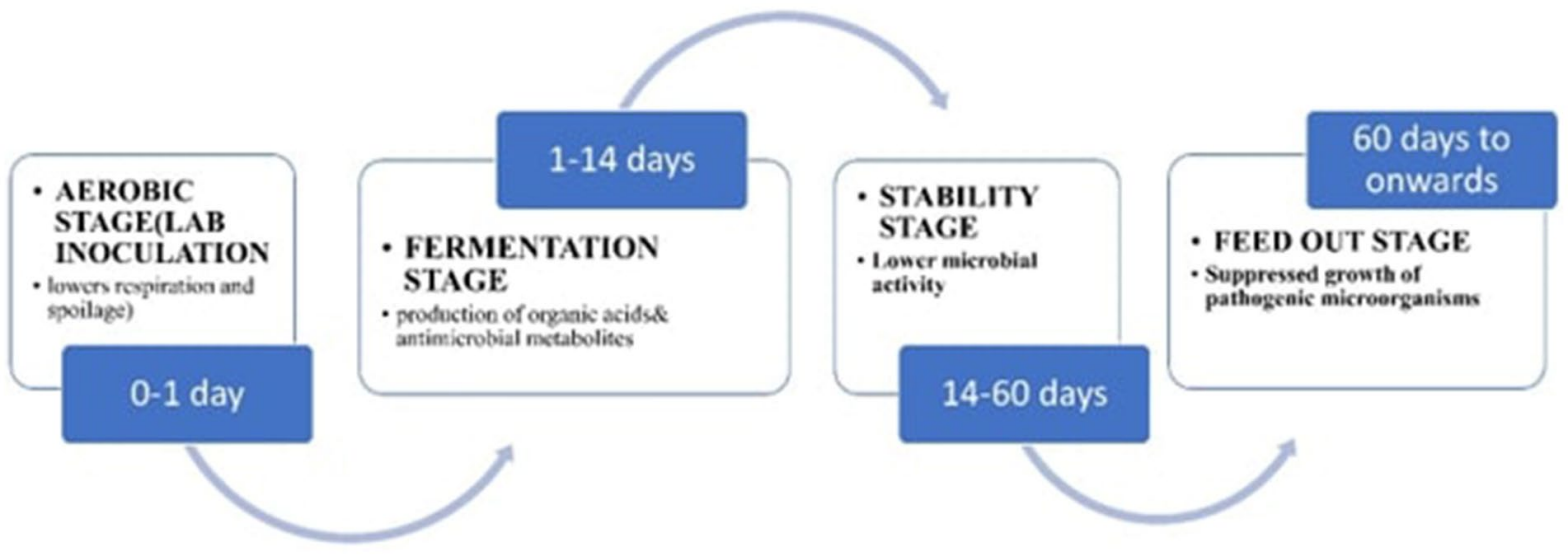
The activity of LAB (Lactic Acid Bacteria) in silage production is governed by four critical biochemical factors: the availability of fermentable sugars, the choice of LAB inoculants, the ability to establish and maintain a low pH under anaerobic conditions, and the type and number of organic acids and metabolites produced.

LAB is crucial for successful ensiling, and numerous studies have documented their role in preserving silage effectively (Contreras-Govea et al., 2011; Rabelo et al., 2019; Bai et al., 2020; Gang et al., 2020). For example, the application of LAB strains such as *L. brevis* and *Furfurilactobacillus parafarraginis* has been shown to improve the consistency of the ensiling process and enhance the overall quality of the silage (Liu et al., 2014; Xu et al., 2017). LAB plays a wide function in silage production, affecting different elements such as pH decrease, dry matter recovery, animal performance, antagonistic activity, changes in the microbial community makeup, and the formation of beneficial organic acids. LAB inoculants are well-known for improving silage quality by promoting acidification (Arriola et al., 2011; Kim et al., 2021). But biomass characteristics like high moisture content, high buffering capacity, high protein content, or low soluble sugar content can also make LAB less effective during the ensiling process. Moreover, LAB safety concerns, decreased activity, and shorter storage times can also have an impact on LAB’s effectiveness (Athmanathan, 2013; Queiroz et al., 2018; Soundharrajan et al., 2021). The activity of LAB in silage production is influenced by four key biochemical factors, as illustrated in Figure 6.

### Acidity level and naturally occurring acid

One fundamental principle of preserving silage is the rapid attainment of a low pH through the process of fermentation (Pahlow et al., 2003). The pH level of ensiled biomass is considered a vital indicator of the effectiveness of the fermentation process (Peng et al., 2021). Table 1 shows the effectiveness in inoculants of LAB in the production of silage. LAB can generate concentrated lactic acid, which is 10–12 times stronger than other organic acids (Kung et al., 2018). In recent research, multiple studies have indicated that pathogens, like yeasts, often play a pivotal role in initiating aerobic spoilage. This is for consumption of lactic acid in aerobic environments, leading to elevated pH levels (Wang et al., 2018). However, adding LAB during the ensiling process quickly reduces the pH of silage in anaerobic conditions, successfully preventing the growth of Clostridia, fungi, yeasts and molds (Muck et al., 2018). Studies have shown that paddy rice silage inoculated with LAB achieved a notably low pH of 3.0, indicating that these inoculants were highly effective for this type of silage (Ni et al., 2016).

Isolating *L. plantarum* and *L. casei* from fruit residues resulteresulted intantial pH reduction in pineapple and papaya peel silages, lowering the pH to 3.4. This reduction significantly developed their long-term preservation for livestock feed (Yang et al., 2016). When evaluating LAB performance in silage preservation, the key factors beyond the pH include production of beneficial organic acids and related metabolites, as highlighted by Kung et al. (2018). Organic acid concentrations, such as lactic and acetic acid, in silage are often inversely related to the contents of dry matter (DM). In superior silages, lactic acid concentrations typically range from 2 to 4% of total DM, although they can be much higher in silages with lower DM content. Many homofermentative LAB strains, such as *L. plantarum, L. acidophilus,* and *L. casei*, are noted for producing significantly high levels of lactic acid in different types of silage (Guo et al., 2013; LI et al., 2018; Jiang et al., 2020). In contrast, silages treated with heterofermentative *L. buchneri* shows the higher levels of acetic acid due to convert of some lactic acid into acetic acid, as demonstrated by Kleinschmit and Kung in 2006 (Kung et al., 2018). Moreover, in various silage acetic acids are the second most common organic acid, usually making up 1 to 3% of dry matter contents.

In addition, the LAB also produces propionic acid, which is usually either undetectable or present in very low concentrations (less than 0.1%) in high-quality silage. It is more commonly found in desiccated or dried-out silage (Kuley et al., 2020). The study found that silage inoculated with *L. plantarum* had a high propionic acid concentration of 63.4 g/kg, which was higher than the concentrations of lactic and acetic acids. Nonetheless, higher concentrations of propionic acid (greater than 0.3–0.5%) in the silages are often associated with poor fermentation. Additionally, the bacteria *Clostridium tyrobutyricum* produces butyric acid, which is commonly found in poorly preserved silages. When butyric acid levels exceed 0.5%, it indicates undesirable clostridial fermentation, which results in a rancid butter flavor that reduces the nutritional value due to the breakdown of soluble nutrients (Kleinschmit and Kung, 2006b). Butyric acid is an indication of clostridial microbe metabolic activity, which causes silages with poor fermentation to lose a lot of dry matter and recover little energy (Pahlow et al., 2003).

Furthermore, Kung et al. (2018) observed that several heterofermentative LAB, enterobacteria, and yeasts create a tiny quantity of ethanol (around 0.5–1.5%), particularly in legume and whole-plant corn silages. Silages that contain ethanol have an alcoholic flavor and are susceptible to aerobic spoiling, which is mostly caused by yeast activity. High ethanol concentrations in silage have been linked to higher dry matter loss and a higher risk of feed poisoning in ruminants (Kung et al., 2018). Furthermore, dairy cows’ systemic blood alcohol levels can be impacted by corn silage, even at low ethanol levels, when they consume it (Kristensen et al., 2007).

### Nutrients composition

LAB is widely recognized for its ability to enhance the quality of silage regarding nutrition during the process of ensiling. While the nutrient profile of silage such as dry matter, crude fiber, carbohydrates, crude proteins, and nitrogen content well-documented, many studies have highlighted the superior nutritional benefits of silage inoculated with LAB compared to untreated silage (Guo et al., 2013; Nazar et al., 2020; Bai et al., 2021). During the production of high-quality silage, that may affect the minimizing dry matter contents is a significant challenge. The addition of LAB during the process of ensiling helps to reduce dry matter loss compared to the substantial losses observed in untreated silages (Kleinschmit and Kung, 2006a). Usually, between 2 and 6% of the dry matter is lost as a result of lactic acid fermentation (Borreani et al., 2018; Villa et al., 2020). Conventionally, LAB reduce ammonia content in silage by accelerating pH reduction, which decreases acetic acid fermentation and enhances dry matter recovery (Ellis et al., 2016). Alfalfa silage inoculated with *L. plantarum* showed a noteworthy increase in dry matter regained. Additionally, the aerobic stability of grass silages improved with rising dry matter content, ranging from 18 to 44%, when inoculated with *L. plantarum* and *L. buchneri*. However, it is important to note that alfalfa silage with high dry matter levels, particularly exceeding 50–55%, may develop a tobacco-like flavor due to heat-damaged proteins resulting from the Maillard reaction (Kung et al., 2018). Carbohydrates include a variety of complex chemical compounds, and their transformation in silages contributes to the reduction of dry matter (Okoye et al., 2023). These carbohydrates are divided into two groups: structural carbohydrates, or those derived from the walls of plants, such as cellulose, hemicellulose, acid detergent fiber (ADF), acid detergent lignin (ADL), and neutral detergent fiber (NDF); and non-structural carbohydrates, or those derived from the contents of plants’ cells (Zhao et al., 2021). Inoculating silage with LAB activates a series of changes that typically signal carbohydrate fermentation (Basso et al., 2012). Homofermentative LAB improves the early stages of the ensiling process by converting the water-soluble carbohydrates (WSC) into lactic acids. For example, during the ensiling process, the addition of *L. plantarum* to corn stover silage resulted in greater amounts of carbohydrates, particularly in NDF. This can offer a valuable roughage source to help alleviate shortages in animal feed (Cai et al., 2020). Nevertheless, LAB that produces lignocellulose enzymes promotes the conversion of plant resources into water-soluble carbohydrates (WSC), making glucose more readily available for subsequent transformation into lactate. This process reduces cell wall carbohydrates, aiding in the effective breakdown of ADF and NDF (Mahdi et al., 2014).

### Aerobic endurance and pathogens management

While silos are opened during the feed-out process, the ensiled material is exposed to air, which starts a deterioration process. This is driven by pathogenic microorganisms, particularly lactate-assimilating yeasts that can survive the initially low pH conditions. As a result, the silage’s pH and temperature rise, which promotes the growth of additional aerobic microorganisms (Kung et al., 2018). Silage’s resistance to deterioration microbes can vary widely, and different LAB species are frequently used to effectively prevent extensive aerobic spoilage (Borreani et al., 2018). Effective silage production methods that use LAB to accelerate the reduction of pH and improve the resistance to aerobic spoilage can help reduce hazards from microbes and chemicals associated with poorly kept silage (Driehuis et al., 2018). Higher concentrations of easily absorbed carbohydrates make foods more prone to aerobic deterioration. For instance, because microbes can swiftly ferment the sugars in crops like corn, sorghum, and sugarcane when oxygen is present, these crops are particularly vulnerable to spoiling (Drouin et al., 2019).

The presence of microorganisms related spoilage, such as *Enterobacteria* and *Clostridia*, speeds up the degradation of silages. However, using LAB inoculants like *L. fermentum* and *L. plantarum* has been shown to effectively address this problem in silages made from corn, oats, and sorghum (Puntillo et al., 2020). Research has shown that adding homofermentative LAB to corn silages and storing at a specific temperature leads to a low yeast count, which is associated with improved aerobic stability. Furthermore, several studies recommend the use of heterofermentative LAB, such as *L. buchneri* and *L. brevis*, to stop pathogen-caused aerobic deterioration in silage (Drouin et al., 2019). Moreover, using a blend of *L. brevis*, *L. plantarum*, and *L. buchneri* resulted in a significant boost in the aerobic stability of corn silage after a 30 days of ensiling (Zhang, 2019). Although *L. plantarum* and *L. buchneri* are frequently used in the ensiling process, some strains of *Bacillus* have also been mentioned to enhance the stability of aerobic conditions of corn silages (Lara et al., 2018). Additionally, numerous studies have shown that LAB produces various antibacterial substances, including bacteriocins, which inhibit the growth of several fungi, such as those in the genera *Monilia, Aspergillus, Penicillium,* and *Fusarium* (Gao et al., 2019).

Many research articles have investigated peptides produced by LAB that display fungi-static properties in the food industry (Özogul and Hamed, 2018). Newly, substances like peptides produced by certain LAB strains, mostly *L. plantarum*, have shown a significant inhibitory effect on the growth of mycelium and spore germination. Additionally, other active metabolites that have been shown to inhibit fungal growth in silages include lauric acid, 10-octadecenoic acid methyl ester, heptadecanoic acid, palmitic acid, stearic acid, and 16-methyl ester (Carrizo et al., 2022). Recent research has focused on the identification of LAB strains that produce unique metabolites to prevent the colonization of pathogenic organisms during the process of ensiling (Kim et al., 2021). For instance, *L. delbrueckii* and *L. plantarum* produces class II-a bacteriocins, which are viewed as promising substitutes to feed antibiotics. These bacteriocins help inhibit pathogenic microorganisms and do not lead to drug resistance in animals after silage inoculation (Li et al., 2020).

### Animal productivity

The use of LAB inoculants in silage production and preservation is driven by their ability to break down lignocellulose, which enhances the digestibility of silage for animals. LAB are widely utilized in animal husbandry because they contribute to a balanced diet, discourage selective feeding behavior, improve digestibility, and stabilize rumen conditions (Contreras-Govea et al., 2011; Muck et al., 2018). Moreover, better growth performance and increased feed efficiency are achieved when LAB is added to silage (Muck et al., 2018). LAB exhibits strong probiotic qualities by surviving in rumen, interacting with other beneficial microorganisms, and maintaining their functionality in animals (Matthews et al., 2019). Silage treated with *L. buchneri* and *L. plantarum* showed improved milk yield and reduced the oxalate impact on diets of cattle.

This resulted in a significant increase in the digestibility of nutrients and overall performance, with no negative effects put on the health of animals. Further, the LAB inoculants, especially homofermentative LAB are associated with better digestibility of nutrients and a decrease in anti-nutritional compounds (Oliveira et al., 2017b). Research has demonstrated that feeding ruminants silage preserved with LAB markedly improves the performance of animals in the rumen environment, leading to better utilization of feed. For example, in-vitro ruminal fermentation studies have shown that LAB-inoculated silages can affect rumen microorganisms, sometimes reduce methane emissions a greenhouse gas and, in some cases, increase microbial biomass production (Muck, 2013).

### Contemporary LAB biotechnology for enhancing silage

Recent advancements in improving silage quality by choosing particular LAB strains designed for given forages is one way to choose LAB inoculants, using LAB that produce lignocellulose degrading enzymes, generating beneficial organic acids and other related metabolites to control the spoilage microorganisms during feed out, and applying the modern molecular techniques such as genomics, metagenomics, metabolomics, proteomics, analysis of gene expression, and cloning of selected strains of LAB. While these approaches have been studied in forage science, their practical application is still somewhat limited (Muck et al., 2018; Nascimento Agarussi et al., 2019). However, the biotechnological potential of LAB in silage production has not yet been fully explored (Amaral et al., 2020).

### Microbiota genomics

While molecular techniques have the potential to transform our comprehension of LAB behavior during ensiling, the accuracy and reliability of the acquired data are contingent on the precision and quality of the nucleic acids that were taken from various sources. Numerous modern methods, including nucleic acid extraction, sequencing, sampling, preservation, and bioinformatics, can be used to determine the makeup and functions of the microbial communities engaged in the ensiling process (McAllister et al., 2018). However, a sample of nucleic acids, which contain a mixture of microbial populations, is compared to their reference genomes through metagenomics to identify and quantify the microorganisms present. Metagenomic sequencing of microbial DNA from a variety of sources, such as recently harvested fodder, is made easier by several platforms, such as Illumina, Roche 454, Ion Torrent, PacBio, and polymerase chain reaction (PCR).(Bao et al., 2016; Duniere et al., 2017). PacBio’s single-molecule real-time sequencing (SMRT) technology effectively revealed the dynamics of microbial communities in alfalfa silages. It provided insights into the prevalence and dominance of LAB homofermenters like *L. plantarum* and heterofermenters like *L. buchneri* (Guo et al., 2018).

Various methods based on PCR have improved the identification of numerous LAB inoculants to enhance quality of silage, as demonstrated by a study conducted by Yu et al. (2020). PCR methods have demonstrated both effectiveness and accuracy in identifying *L. buchneri* in silage samples. Several metagenomic methods have been employed in the last ten years to track changes in microbial communities and identify common species, including denaturing gradient gel electrophoresis (DGGE) and terminal restriction fragment length polymorphism (TRFLP). Additionally, 16S rRNA has been used in several investigations to categorize LAB populations in silage (Schmidt and Kung, 2010). For example, 16S rRNA sequencing enabled the identification of highly effective LAB strains in the silages made from perennial ryegrass, corn, alfalfa, and sorghum, which were then used to inoculate alfalfa silage. Meta-genomic techniques provide a detailed understanding of microbial ecology during the ensiling process, including how epiphytic microorganisms affect the quality of silage and how these dynamics can be effected by LAB inoculants (Nazar et al., 2020). Until now, there has been no effort to combine the advantages of PCR-based profiling technologies like PCR-DGGE with next-generation sequencing (NGS).

Furthermore, it has been shown that employing primers that target less variable areas of ribosomal DNA or other genes as opposed to universal primers may yield more accurate findings and raise the species-level similarity scores (Drouin et al., 2019). Nonetheless, incorporating metagenomics to better understand high-performing LAB strains will refine and improve the process of ensiling.

### Analyses of genome

A thorough analysis of the entire genome is key for the identifying of useful organic acids and secondary metabolites. Systems biology, which encompasses metagenomics, transcriptomics, proteomics, and metabolomics, continues to rely heavily on genetic data to get a deeper comprehension of the distinctive features of living animals (Liu et al., 2014). For instance, complete genome sequencing of *L. hokkaidonensis*, a psychrophilic LAB strain isolated from grass silage, provided valuable insights into the genetic makeup and evolutionary history of this specific group of LAB (Tanizawa et al., 2015). Additionally, a genome mining tool has been created to examine the potential genome of bacteriocin clusters with antagonistic properties.

However, due to the short length and variability sequence of peptides involved in the bacteriocin synthesis, interpreting the open reading frames (ORFs) related to the bacteriocin production can be challenging. These ORFs are often found alongside many genes involved in genome regulation and transport (Pfeiler and Klaenhammer, 2007). These genes enable the mapping of entire metabolic pathways and the utilization of intriguing aspects to enhance the quality of silage (Bintsis, 2018). Moreover, detailed re-sequencing of lateral genome has opened up new opportunities in field of applied genomics, especially for the characterizing of novel species with the bio-preservative potential (Douillard and De Vos, 2014).

### Transcript profiling

Developments in transcriptomics have enabled the investigation of the functional effects of genetic diversity. Modern technologies, such as sequencing of RNA and high throughput next-generation sequencing (NGS), have become leading methods for studying transcriptome (Wang et al., 2018). Moreover, like genomics, the transcriptomics is influenced by environmental factors (temporal and cellular), allowing for the examination of different stages in the cell life cycle, the interpretation of genome functions, the prediction of molecular components, and the exploration of various biological processes (Gu and Zhao, 2019). Today, transcriptomics has become an essential method for in-depth exploration of biological gene expression patterns, proving more effective than genome-level analysis (genomics). For instance, the recent sequencing of the *L. buchneri* genome has provided a critical resource for identifying significant genetic factors in the ensiling process. Genes with differential transcription were used to predict the LAB strain’s potential for improving silage quality (Eikmeyer et al., 2013). Various LAB species have been characterized through DNA micro-arrays, with their transcriptomic profiles overlaid onto metabolic maps. This method allows for the identification of gene expression patterns associated with metabolic pathways. Such insights can reveal the expression of genes that encode enzymes involved in producing and metabolizing beneficial organic acids, proteolysis, or mycotoxin generation during the ensiling process (McAllister et al., 2018).

### Protein expression analysis

Proteomics involves a comprehensive examination of proteins, which are fundamental cellular constituents or biomolecules, as well as other secondary metabolites. This study encompasses their structure and their physiological functions (Niyitanga et al., 2022). Proteomics aims to assess various aspects of proteins, such as their localization, isoforms, abundance, posttranslational modifications, and molecular interactions. It is also used to identify proteins in bacterial systems. In the context of LAB, proteomics has been used to determine cell surface proteins, map protein content, characterize LAB responses to different conditions of fermentation, and explore molecular biology (Vinusha et al., 2018). Proteomics has developed into a multidisciplinary field that integrates biology, physics, chemistry, bioinformatics, and computer science, emphasizing high throughput techniques and minimizing user bias.

Technology can differ, but they typically combine techniques including isolation, separation, detection, and identification. These techniques include matrix-assisted laser desorption/ionization time-of-flight mass spectrometry (MALDI-TOF-MS), reverse-phase high-performance liquid chromatography (RP-HPLC), mass spectrometry (MS), liquid chromatography-mass spectrometry (LC/MS), and sodium dodecyl sulfate-polyacrylamide gel electrophoresis (SDS-PAGE) (Lubeckyj, 2021). Recent research has utilized proteomics technologies to examine LAB strains that produce helical amphiphilic protein metabolites, such as bacteriocins, which are recognized for their preservative properties in silage production (Darvishi et al., 2021). For instance, plantaricin LPL-1, a novel bacteriocin produced by *L. plantarum* with a molecular mass of 4347.85 Da, was purified, identified, and quantified using methods like gel filtration, matrix-assisted laser desorption/ionization time-of-flight mass spectrometry (MALDI-TOF-MS), and RP-HPLC. Furthermore, strains with high acidification rates and advantageous preservative qualities have been found by bioactive proteins and antimicrobial peptides from silage, which successfully prevent the growth of *S. aureus* and *E. coli* while enhancing the quality of paddy rice silage (Ni et al., 2015b; Gavrilova et al., 2019).

### Metabolomics

Metabolomics is a branch of omics, that studies the range of naturally occurring molecules having low mass in biological contexts and fields. The metabolomic analysis includes two primary approaches: targeted metabolomics, which aims to quantify a specific set of distinct molecules, and untargeted metabolomics, which seeks to identify and measure a broad spectrum of metabolites with varied characteristics (Okoye et al., 2023). Advancements in bioinformatics and analytical technologies, together with the integration of multiple biological techniques, have broadened the scope of metabolomic analyses, allowing for a comprehensive understanding of the systemic effects of metabolites. Additionally, the great specificity of metabolomics enables the detection of minor changes in metabolic pathways, providing vital insights into the mechanisms behind diverse physiological states and abnormalities, including those generated by pathogens (Johnson et al., 2016). Modern metabolomic technologies can precisely analyze a broad range of metabolites, surpassing the capabilities of traditional analytical chemistry methods (Clish, 2015).

Metabolomics commonly utilizes well-established analytical techniques such as mass nuclear magnetic resonance (NMR) and spectrometry (MS), often combined with separation methods like high-performance liquid chromatography (HPLC) or ultra-performance liquid chromatography (UPLC) gas chromatography (GC), and capillary electrophoresis (CE) (Emwas et al., 2019). Profiling the silage microbiota using metabolomics can deepen our understanding of the various biological processes involved in silage production (Xu et al., 2019). For example, extensive information about the metabolites and related activities of silage inoculated with several LAB strains was obtained using gas chromatography–mass spectrometry (GC–MS) (Guo et al., 2018).

### Genetic engineering

Genetic manipulation, commonly known as genetic engineering, is a key technique for introducing changes by activating new genes (Ashery et al., 2014). Hence, it is essential to optimize the utility of LAB, as they have the potential to enhance silage quality, influencing factors such as taste, consistency, and biopreservation (Papagianni, 2011). In the past ten years, genetic manipulation techniques, such as genetic transformation and genome editing, have rapidly advanced, demonstrating impressive versatility across a wide range of fields, from fundamental research to practical applications in biotechnology (Li et al., 2020). While a significant number of LABs are unable to directly ferment plant lignocellulose biomass, they have shown success when engineered, and they serve a unique function in contemporary biotechnology. This is particularly evident when they are co-cultivated with native cellulolytic microorganisms, potentially reducing the expense associated with cellulase additives (Tarraran and Mazzoli, 2018). For instance, in high-moisture silages, Lactics strains modified to generate cellulase enhanced the quality of fermentation and accelerated the breakdown of both nonstructural and structural carbohydrates (Liu et al., 2019).

Understanding how to engineer strains of LAB for modern product and application development is essential. Genetic engineering is crucial for obtaining new insights and discovering unique traits, containing the incorporation and integration of foreign genes into the genome of LAB (Börner et al., 2019). For instance, a maximum genetic transformation efficiency of 5.7 × 10^3^ transformants was achieved when plasmid vectors were electroporated into *L. pentosus* to produce silage. Moreover, the extracted DNA plasmid showed no rearrangement or deletions (Ma et al., 2020). Furthermore, when evaluating preservative properties of electroporated *L. plantarum* in grass silages, it is clear that genetically modified strains of *L. plantarum* can proliferate and outperform native LAB, thereby improving silage quality (Fabiszewska et al., 2019). However, there has been limited research focused on modifying LAB metabolic pathways to adjust cellular metabolism, which would facilitate the efficient production of desired compounds through metabolic reactions (Gordy and Goller, 2020). For example, disrupting the D-lactate dehydrogenase gene through the integration of chromosomes through genetic transformation resulted in the production of L-lactic acid at levels comparable to those of the unmodified wild-type strain of *Lactobacillus. helveticus* (Hatti-Kaul et al., 2018). Genome editing copies natural DNA repair by using gene knockout and knock-in techniques in LAB. New developments in genome editing instruments, like zinc-finger nucleases (ZFNs) CRISPR-Cas9 nucleases, and transcription activator-like effector nucleases (TALENs), have accelerated the shift from the theoretical concepts to practical industrial applications, aimed at improving the probiotic characteristics of LAB (Wu et al., 2021). By introducing different genomic changes, such as insertions, deletions, inversions, duplications, translocations, and point mutations, these approaches have been used to modify native genes. This provides researchers with the technical tools needed to carry out genetic manipulations effectively (Li et al., 2020). Significant progress has been made in the field of genome editing of LAB using CRISPR-based technologies. This method makes it easier to quickly generate mutant strains, which in turn speed up basic research and practical applications. For instance, it has made it possible to generate highly efficient small deletions (<1.0 kb and <100 bp) marker-free, with rates as high as 100% in *Limosilactobacillus reuteri* and *L. lactis*, respectively. In addition, it has made it possible to delete and insert markers at rates of 25 to 65% in *L. casei*, as well as precisely target and suppress multiple genes in Lc. lactis and *L. plantarum* (Song et al., 2017; Guo et al., 2018).

### Modern biotechnological methods

The most successful LAB strains can be identified by using metagenomics and genomics to fully identify and characterize microbial communities and their genetic potential in silage. By analyzing fermentation end products, metabolomics makes it possible to evaluate the quality of silage and the effects of various LAB strains (Carvalho et al., 2021; Okoye et al., 2023; Du et al., 2024). Understanding LAB metabolic pathways and improving strain selection are made possible using transcriptomics and proteomics, a technique that offers data on gene and protein expression during fermentation (Okoye et al., 2023). To improve silage, LAB strains with desired features are developed or enhanced by genetic manipulation (Okoye et al., 2023).

### LAB strains best for silage production

Strong acid tolerance improved lactic acid content, decreased pH, improved silage quality, and improved flavor are all attributes of *L. plantarum*, which is prominent in well fermented silage (Carvalho et al., 2021; Soundharrajan et al., 2023; Du et al., 2024). High lactic acid is produced, pH is lowered, fungi are suppressed, silage quality is enhanced, and *Ligilactobacillus sali*var*ius* AS22 shows probiotic potential (Balasubramanian et al., 2021). *L. hilgardii* (UFLA SIL51, SIL52) is good for sugar cane silage because it minimizes butyric acid, ethanol, and dry matter loss (Balasubramanian et al., 2021). Compared to single strains, mixed LAB cultures yield higher levels of lactic acid and nutritional value (Soundharrajan et al., 2021).

### Applications and selection

LAB inoculants are selected based on their capacity to control fermentation, inhibit unwanted microorganisms, and improve the quality of nutrients. The quality of silage can frequently be improved by mixed LAB cultures more effectively than by single strains (Soundharrajan et al., 2023). For best outcomes, selection should consider local conditions and the crop substrate (Carvalho et al., 2021).

### LAB strains mechanisms and effects

Rapid lactic acid production by *L. plantarum* results in a rapid pH decrease, enhanced fermentation properties, and the inhibition of pathogenic bacteria, molds, and yeasts that cause spoiling (Zhang et al., 2016). By converting lactic acid to acetic acid, which inhibits yeasts and molds, *L. buchneri* is particularly effective at improving aerobic stability and preventing spoiling when silage is exposed to air (Muck et al., 2018). The advantages of both strains can be obtained by using combination inoculants (*L. plantarum + L. buchneri*), which can produce both quick acidification and sustained aerobic stability (Li et al., 2024).

### Inhibition of undesirable microorganisms

These LAB strains produce safer, higher quality silage by lowering the number of spoiling organisms (such as *Clostridium, Bacillus*, *Enterobacteriaceae*, and other fungi). The suppression of undesirable microbial development is largely dependent on lower pH and higher concentrations of organic acid (Mu et al., 2022; Wu et al., 2023).

### Patterns and prospects

Currently, there are numerous obstacles and restrictions associated with using LAB inoculants in the production and storage of silage. Primarily, temperature and moisture levels are key factors affecting the quality of silage. In colder climates, the advantages of LAB inoculants for silage are significantly reduced because low ambient temperatures inhibit the biological activity of the inoculants, leading to decreased fermentation levels in the silage (Campbell et al., 2020; Mejía-Avellaneda et al., 2022). A lot of LAB strains work well for fermenting biomass from crops or fodder that has a high water-soluble carbohydrate (WSC) content, low buffering capacity, and relatively low moisture content. However, some forages, like alfalfa, have high moisture levels and buffering capacity. These forages require LAB inoculants that can rapidly ferment the biomass to quickly lower the silage pH and inhibit pathogen growth. Additionally, LAB inoculants that produce lignocellulolytic enzymes, such as cellulase, xylanase, and laccase, can increase the WSC content in grass silage (Guo et al., 2018; Hu et al., 2020). Therefore, there is an urgent need to develop new LAB strains with specific fermentation traits. Traditional methods for creating unique LAB strains by techniques like laboratory evolution and random mutagenesis, or by removing them from natural sources, are often inefficient and time-consuming. In contrast, genome editing techniques are becoming increasingly popular for improving LAB strain genetics due to their greater success rates, ease of use, and safety.

Additionally, it is essential to recognize that silage toxins can cause severe illnesses with high fatality rates in both humans and animals. During the ensiling process, these toxins are linked to the proliferation and metabolic activities of pathogenic microbes such as *E. coli, Bacillus cereus, Clostridium botulinum, Listeria monocytogenes,* yeasts, and molds. Furthermore, the presence of toxins in plant tissues at harvest that can persist through the process of ensiling is a significant concern (Driehuis et al., 2018). The growth of these pathogens leads to significant spoilage issues, such as stains, disagreeable smells, strange tastes, slime, and other undesirable physical as well as chemical changes that make the silage unappealing. Thus, it is imperative to create LAB inoculants that can control these pathogens’ growth and development throughout ensiling. The outcomes of physical binding to bacterial cell walls and conventional fermentation techniques are usually moderate and have limitations. To enhance this, the utilization of transcriptomics, metabolomics, and genetic engineering can yield a significant understanding of the routes and mechanisms of action. This approach offers the potential to enhance key genes involved in producing the beneficial metabolites in LAB, thereby helping to control the pathogen growth during the process of ensiling.

Moreover, LAB strains with varying fermentation traits have distinct effects on silage quality. A poor-quality silage fermentation is often related to the production of nitrogenous compounds, biogenic amines, and excessive butyric acid. Creating mixed LAB inoculants is becoming interesting, which generally provide better results for silage quality than single inoculants. While some LAB inoculants have shown potential, our current understanding of the fermentation and regulatory mechanisms of different LAB strains in the context of silage is still quite limited (Ellis et al., 2016; Xu et al., 2017; Borreani et al., 2018). Multiomics approach can be applied to explore changes in microbial communities, metobolite profiles, and genes expression levels of LAB. This strategy provides a critical pathway for uncovering the mechanisms that drive interactions among LAB species during silage production and preservation. Additionally, it may help in the development of more effectively combined LAB inoculants with enhanced precision.

## Conclusion

Silage preservation involves ensiling fresh forage crops or other types of biomasses for future use. The quality of silage is improved by incorporating various LAB inoculants, which are effective throughout fermentation, storage, and feed-out phases. Modern biotechnological tools, including metagenomics and metabolomics, provide understandings into silage fermentation, allowing for the identification and production of improved LAB strains. *L. plantarum*, *L salivarius*, and other mixed LAB cultures are sublime alternatives for making high quality silage. *L. plantarum, Pediococcus acidilactici, P. pentosaceus and L. brevis* and *L. buchneri* are the most essential strains for decreasing pH, promoting fermentation, increasing aerobic stability, and suppressing unwanted microbes in silage for animal feed. Using these strains separately or in combination improves silage quality and safety.
